# Supplementary material for: The Evolution and Characterization of the RNA Interference Pathways in Lophotrochozoa
Source: Genome Biol Evol. 2024 May 7;16(5):evae098. doi: 10.1093/gbe/evae098 (PMC11114477; doi:10.1093/gbe/evae098)
Supplement: evae098_Supplementary_Data [file evae098_supplementary_data.zip › Supplementary_materials.zip!/selected_miRNAs/NoGe_selected_miRNAs/known_miRNA_families_pdf_files/NMRB01000320.1_Notospermus_geniculatus_strain_Ushimado_scaffold320,_whole_genome_shotgun_sequence_10298.pdf]

Provisional ID	:	NMRB01000320.1_Notospermus_geniculatus_strain_Ushimado_scaffold320,_whole_genome_shotgun_sequence_10298
Score total	:	37685.4
Score for star read(s)	:	3.9
Score for read counts	:	37676.7
Score for mfe	:	1.7
Score for randfold	:	0
Score for cons. seed	:	3
Total read count	:	73913
Mature read count	:	73389
Loop read count	:	0
Star read count	:	524

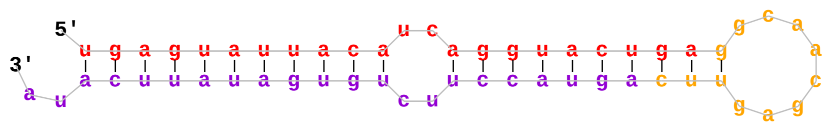

Genome coordinates: NMRB01000320.1 437736-437792

pre-miRNA sequence:
UGAGUAUUACAUCAGGUACUGAUGAGUAUUACAUCAGGUACUGAGGCAACGAGUUCAGUACCUUCUGUGAUAUUCAUA

Mature sequence: UGAGUAUUACAUCAGGUACUGA

Aligned mature sequence of the *N. geniculatus* pre-miRNA with the top 5 hits from the BLAST search against the pre-miRNA mirGeneDB set:

```
                              **********************
Nge_Mir-12                    ugaguauuacaucagguacuga
Npo-Mir-12_5p                 ugaguauuacaucagguacuga
Ovu-Mir-12_5p                 ugaguauuacaucagguacuga
Cte-Mir-12_5p                 ugaguauuacaucagguacuga
Lgi-Mir-12_5p                 ugaguauuacaucagguacuga
Cgi-Mir-12_5p                 ugaguauuacaucagguacuga
```
